# Heuristic Identification of Biological Architectures for Simulating Complex Hierarchical Genetic Interactions

**DOI:** 10.1002/gepi.21865

**Published:** 2014-11-13

**Authors:** Jason H Moore, Ryan Amos, Jeff Kiralis, Peter C Andrews

**Affiliations:** Department of Genetics, Institute for Quantitative Biomedical Sciences, Geisel School of Medicine, Dartmouth CollegeHanover, NH, United States of America

**Keywords:** epistasis, simulation, bioinformatics, software

## Abstract

Simulation plays an essential role in the development of new computational and statistical methods for the genetic analysis of complex traits. Most simulations start with a statistical model using methods such as linear or logistic regression that specify the relationship between genotype and phenotype. This is appealing due to its simplicity and because these statistical methods are commonly used in genetic analysis. It is our working hypothesis that simulations need to move beyond simple statistical models to more realistically represent the biological complexity of genetic architecture. The goal of the present study was to develop a prototype genotype–phenotype simulation method and software that are capable of simulating complex genetic effects within the context of a hierarchical biology-based framework. Specifically, our goal is to simulate multilocus epistasis or gene–gene interaction where the genetic variants are organized within the framework of one or more genes, their regulatory regions and other regulatory loci. We introduce here the Heuristic Identification of Biological Architectures for simulating Complex Hierarchical Interactions (HIBACHI) method and prototype software for simulating data in this manner. This approach combines a biological hierarchy, a flexible mathematical framework, a liability threshold model for defining disease endpoints, and a heuristic search strategy for identifying high-order epistatic models of disease susceptibility. We provide several simulation examples using genetic models exhibiting independent main effects and three-way epistatic effects.

## Introduction

Sorting out the genetic architecture of common human diseases will require a combination of computational, mathematical, and statistical methods that are capable of detecting, characterizing and interpreting a wide range of different genetic effects including gene–gene interactions, gene–environment interactions and locus heterogeneity [Cordell, 2009; Moore et al., 2010]. A key component of methodological work is the ability to know when a particular method is working well. Application of quantitative methods to real data provides useful information but is not sufficient due to the lack of knowledge about the true underlying patterns. Simulation of genetic data is thus an important component of the method development process because the ground truth in the data is always known. The primary disadvantage of simulation is the inability to know the exact nature of genetic models that underlie the genetic architecture of complex diseases. Given this constraint, an important goal of simulation is to incorporate as much knowledge as possible about genetic architecture to improve the realism and complexity of the data needed for testing new methods.

There are two general components to the simulation of genetic epidemiology data. The first is the simulation of realistic patterns of genetic variation that mirror the distribution of allele frequencies and linkage disequilibrium that occur in human populations. The second is the simulation of complex traits from particular genetic variants. Numerous methods have been developed for the first component including forward-time simulators such as simuPOP [Peng and Amos, 2008; Peng and Kimmel, 2005], GenomeSIMLA [Dudek et al., 2006; Edwards et al., 2008; Ritchie and Bush, 2010], and SFS_CODE [Hernandez, 2008]. Once realistic patterns of genetic variation have been simulated, the next step is to select a set of genetic variants and a model from which phenotypes can be generated for each subject in the data set. This can be accomplished, for example, using a linear model with an effect size determined by the magnitude of the coefficients for the independent variables representing the selected genetic variants. An alternative approach is to use penetrance functions that specify the probability of disease given a particular genotype or combination of genotypes. Several software packages are available to simulate complex genetic patterns (e.g. epistasis) from penetrance functions including Epi2Loc [Walters et al., 2014] and GAMETES [Urbanowicz et al., 2012]. Although useful, these tools, and other commonly used phenotype simulation tools such as PLINK [Purcell et al., 2007], lack a biology-based framework. That is, these tools are purely statistical in nature and do not integrate knowledge about gene structure and function into the simulation. It is our working hypothesis that the simulation of data using biologically realistic genotype–phenotype relationships will improve method development by more closely mimicking the hierarchical complexity of human health.

The goal of the present study was to develop a prototype genotype–phenotype simulation method and software that are capable of simulating complex genetic effects within the context of a hierarchical biology-based framework. Specifically, our goal is to simulate multilocus epistasis or gene–gene interaction where the genetic variants are organized within the framework of one or more genes, their regulatory regions and other regulatory loci (e.g. microRNA). Simulating data in this manner is important because the genomic context of genetic risk factors is becoming increasingly apparent as we analyze and interpret polymorphisms identified through genome-wide association studies [ENCODE Project Consortium et al., 2012; Gerstein et al., 2012; Karczewski et al., 2013]. We introduce here the Heuristic Identification of Biological Architectures for simulating Complex Hierarchical Interactions (HIBACHI) method and prototype software for simulating data in this manner. This approach combines a biological hierarchy, a flexible mathematical framework, a liability threshold model for defining disease endpoints, and a heuristic search strategy for identifying high-order epistatic models of disease susceptibility. We provide several simulation examples using genetic models exhibiting independent main effects and three-way epistatic effects.

## Methods

There are five components to our Heuristic Identification of Biological Architectures for simulating Complex Hierarchical Interactions (HIBACHI) simulation method. The first is the biological framework. The second is the mathematical framework. The third is the liability threshold model. The fourth is the heuristic methods for the discovery of high-order epistasis models. The final component is the prototype software package for simulating multiple data sets. We describe each of these in turn.

### A Biology-Based Framework for Genetic Simulation

**Figure 1:**
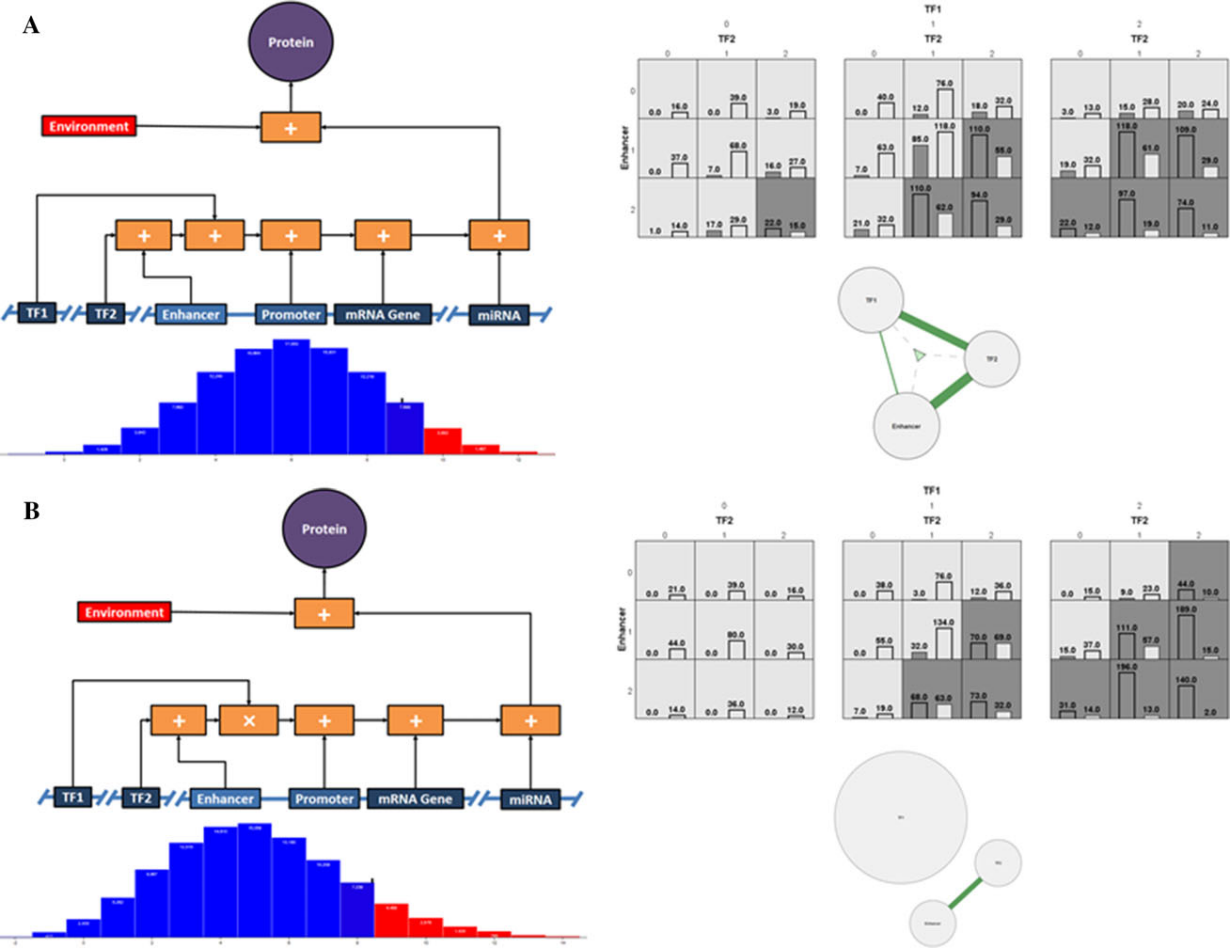
The left panels show screenshots of the biological and mathematical framework as well as the liability distribution for Models 1 (A) and 2 (B). The black notches on the right side of the liability distributions indicate the threshold for disease. To the right of each HIBACHI model is the MDR model showing the distribution of cases (dark bars) and controls (light bars) for each genotype combination. Dark-shaded genotype combinations indicate high-risk of disease. Shown below each MDR model is the ViSEN network with main effects (circles), pairwise interactions (lines), and three-way interactions (triangles) highlighted in proportion to their effect size.

The goal of this component is to provide a framework or scaffold for organizing the genetic variants and their phenotypic relationships. Although not complete, our approach is a step in the direction of using known biological relationship to focus a simulation. Our initial framework (see Fig.1) starts with protein-coding gene (i.e. mRNA gene) with a single nonsynonymous genetic variant that is assumed to change an amino acid. Upstream of the mRNA gene is a promoter with a single regulatory variant and an enhancer with a single regulatory variant. Also included in our initial framework are two genes that code for transcription factors that bind to the regulatory region. We have included a protein-coding variant in the gene that codes for each transcription factor. We have also included a single variant in a microRNA gene that participates in post-translational regulation. In total, this structure allows for six genetic variants (coded 0, 1, 2) all influencing a protein product as a quantitative trait. In addition, we have included an environmental factor (coded −2, −1, 0, 1, 2) to allow for nongenetic variation in the phenotypic values. It is important to note that this particular biological framework is a preliminary proof of concept and will be modifiable by the user in future iterations of the algorithm.

### A Mathematical Framework for Genetic Simulation

The goal of this component is to provide a flexible mathematical framework for combining genotypic and nongenotypic values to produce phenotypic values. Each biology-based locus feeds into a mathematical function whose result is carried forward to the next function (see Fig.1). For example, one transcription factor locus combines with the enhancer locus through a function whose result then combines with the second transcription factor. The result of this operation combines with the locus at the promoter. This result combines with the coding variant in the gene. This result combines with the microRNA locus. This result combines with the environmental factor to produce a protein product. Thus, the protein expression value is dependent on mathematical functions of the six loci and the environmental factor. This produces a discrete distribution with several to millions of possible phenotypic values for most combinations of functions that can then be used with the liability threshold model described below to generate disease status. Cases and controls can then be sampled from this distribution.

At each combination of loci the user can specify one of 25 different mathematic functions organized into groups labeled Basic, Logical, Bitwise, Unary, Large, and Miscellaneous. Basic functions include addition (ADD or +), subtraction (SUB or −), multiplication (MULT or ×), division (DIV or ÷), modulus (MOD), and modulus-2 (MOD2). Logical functions include greater than (GT or >), less than (LT or <), AND (&&), OR (∥), and XOR (^^). Bitwise functions include bitwise AND (BITA or &), bitwise OR (BITO or |), and bitwise XOR (BITX or ⊕). Unary functions include absolute value (ABS), NOT (∼), factorial (FAC or !), left and right. Large functions include power (POW), log, permute (PER or P), and choose (CHS or C). Miscellaneous functions include minimum (MIN) and maximum (MAX). These provide a comprehensive array of possible mathematical functions with 25^6^ function combinations using the biological framework described above.

### A Liability Threshold Model for Biology-Based Genetic Simulation

We have used a liability threshold model to simulate disease from the distribution of phenotypic values generated from the genotypic values and mathematical functions as described above (see distribution in Fig.1). The user can select the liability threshold to achieve a particular disease prevalence. HIBACHI generates liability distributions with diverse applications and interpretations. For instance, by selecting ordinary addition for all the mathematical functions the approach gives the liability function for independent, additive genetic variants. At the opposite end of the spectrum, it can model purely epistatic interactions among the variants by choosing the mathematical functions suitably. A wealth of other applications are possible using the 25^6^ choices for the mathematical functions. Given a choice of functions, the liability distribution from which the output of HIBACHI effectively samples can, in theory, always be computed mathematically, though the amount of computation required may be prohibitive. For instance, if all the functions are addition the output is obtained by sampling, once for each population member, from the distribution equal to the sixfold convolution product of the probability distributions *A_i_* of the six genetic factors. Specifically, if *x_ij_* are all the values of factor *A_i_*, the value of this distribution at *x* is

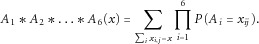
1

This is the sum of the probabilities of all the ways that the output could equal *x*. With all functions as addition, the output is *x* when the attributes have values which sum to *x*.

Thus the sum in (1) is taken over all attribute values *x_ij_* which sum to *x*. HIBACHI chooses values of genetic factors independently, so the joint probability that the six genetic factors have specific values (summing to *x* for instance) is easily computed:

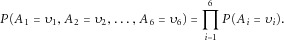


Hence the product of probabilities in (1).

For choices of functions other than all addition, the distribution can be computed as in (1) with one change: Rather than summing over all values which sum to *x*, the sum is taken over all values which combine as prescribed by the functions to give *x*.

As an example we show how HIBACHI can model epistatic interactions. Let us assume each genetic variant has genotypes coded 0, 1, 2 that occur with probabilities 0.25, 0.5, and 0.25, respectively. Thus if the first function is MOD2 and the value of just the first genetic variant is known, then a little computation shows that the result of the first function is either 0 or 1, each occurring with equal probability. Similarly if the value of the second genetic variant is known, but not the first, the output of the first function is again 0 or 1 with equal probability. However, if the values of the first and second genetic variant are both known, then the output of the first function is completely determined. Thus the choice of XOR, a known epistasis function, for the first function generates a synergistic interaction between the first two genetic variants. Choosing from the other functions either left or right, as appropriate, from the Unary menu of HIBACHI passes the result of the first function and this epistatic behavior to HIBACHI's final output. With these choices, data sets generated by HIBACHI have the property that each of the first two genetic variant alone convey no more information about the output than guessing, whereas their values together completely determine the output.

### Heuristic Methods for Model Discovery

The 25^6^ possible combinations of mathematic functions are too many to explore exhaustively by trial and error. We have therefore provided two initial stochastic search algorithms to facilitate this process. The first is a simple random search that will explore *n* randomly generated models where *n* is specified by the user. The second is a genetic algorithm that evolves a population of bitstrings specifying combinations of six mathematical functions. We have previously used an approach like this to evolve penetrance functions specifying two-way to five-way epistatic effects [Moore et al., 2004]. With the genetic algorithm, a population of bitstrings of size *m* is randomly initialized and evaluated using a fitness function (described below). The best bitstrings are mutated and recombined to generate new bitstrings that generate a new population. This process of evolving combinations of mathematical functions using natural selection is carried out for *l* generations allowing the algorithm to explore a maximum of *n* = *m* × *l* possible models. Other stochastic search algorithms such as simulated annealing will be added at a later time giving the user several different options for the heuristic search. These will be important as the complexity of the simulation grows in future versions that allow additional genetic variants influencing additional genes and regulatory elements.

The key to the genetic algorithm search is the fitness function that specifies the value or quality of a particular set of mathematic functions represented as a bitstring. Here, we used an entropy-based method that measures the pure three-way epistatic interaction after subtracting out the one-way and two-way genetic effects [Hu et al., 2011; Hu et al., 2013a,b]. This approach has been used to describe three-way interactions in genetic epidemiology studies of bladder cancer [Hu et al., 2013] and tuberculosis [Collins et al., 2013; Hu et al., 2013; White et al., 2014]. This implementation requires a single data set to be simulated from the model specified by the bitstring from which the three-way interaction information is calculated. Other measures of genetic association will be added to the fitness function list in future versions allowing the user to specify the nature of the genetic effects to be modeled.

### A Prototype HIBACHI Software Package

The goal of the software development was to produce a prototype that could be used to evaluate the method and to generate ideas for the design of a formal software package for public release. The HIBACHI prototype was programed in Java and Javascript with a graphic user interface (GUI) that allows the user to specify the mathematical functions for each point in the biological hierarchy (see Fig.1) in addition to the details of the model discovery heuristic as well as the details of the simulation parameters. Users can specify simulation parameters including the population size, disease prevalence, the sample size, the balance of cases, and controls, the number of randomly generated polymorphisms to add along with their range of allele frequencies, and the number of datasets to be generated. Also included in the GUI is a table that includes all of the models discovered over time along with their accuracy from the multifactor dimensionality reduction (MDR) machine learning classifier [Hahn et al., 2003; Moore, 2010; Moore et al., 2006; M D Ritchie et al., 2001] and the three-way interaction information score as described above. The models are provided in a ranked list that allows the user to select a specific model to base a simulation on. The prototype software is available from the authors upon request.

### Generation and Evaluation of HIBACHI Models

The goal of evaluation was twofold. First, we wanted to confirm that HIBACHI is capable of producing genetic models with simple independent main effects and more complex epistatic interactions. To simplify the evaluation we varied and restricted analysis of the genetic models to only the three variants in from the two transcription factor genes and the enhancer. The other genetic and nongenetic factors were fixed as additive effects but subsequently ignored as if they had not been measured in a genetic study. As a baseline we generated genetic models using only the addition (+) functions (Model 1), one addition and one multiplication (X) function (Model 2), and two multiplication functions (Model 3). We then generated a similar model using only the XOR (^^) logic function (Model 4) that is known to generate statistical epistasis effects [Moore et al., 2006; Moore et al., 2005] and that has a biological basis [Buchler et al., 2003; Tagkopoulos et al., 2008].

The second goal was to use the heuristic genetic algorithm to discover new models exhibiting three-way epistasis for the genetic variants defined above from among the 25^6^ possible combinations of mathematic functions. We ran the genetic algorithm with a population size of 100 for 100 generations. This run represents a search of a maximum of 10^4^ of the 25^6^ possible models. The four best models were recorded and compared with Models 1–4 for strength of three-way interaction. It is important to note that we altered the mathematical functions for the remaining genetic loci and the environmental factor to match Models 1–4. The new models discovered represent Models 5–8.

We then simulated data for 1,000 cases and 1,000 controls from a total population size of 100,000 subjects with a common disease prevalence set to 0.10. Here, we assumed each locus had two alleles of equal frequency with genotype frequencies consistent with those expected under Hardy-Weinberg equilibrium. Example data sets from Models 1–8 were first analyzed using the MDR machine learning method and software package that was designed specifically for detecting and characterizing high-order gene–gene interactions. The three-locus MDR models were returned along with the accuracy of classification. In addition, we performed two different permutation tests to validate the significance of the model. First, we performed a standard permutation test of the null hypothesis of no association by randomizing the case-control labels 1,000 times. Second, we performed an explicit test of epistasis [Greene et al., 2010] that specifically tests the null hypothesis that the only genetic effects in the model are independent by randomizing genotypes within each genetic variant and within cases and controls separately. This preserves the allele and genotype frequency differences while randomizing the interactions. We also used the ViSEN method and software for visualizing gene–gene interaction networks and for estimating genetic effects using methods from information theory [Hu et al., 2013a; Hu et al., 2013]

Finally, we ran several popular machine learning algorithms to illustrate the utility of the HIBACHI simulations for comparing different methods. We selected only the functional variants from the example data sets described above for Models 1–8. Machine learning methods included naïve Bayes (NB), classification trees (CT), k-nearest neighbors (kNN), neural networks (NN), and support vector machines (SVM). The NB approach used a relative frequency prior with a LOESS window of 0.5 and 100 sample points. This method reflects the main effects in the data. The CT approach required at least five data points in each leaf of the tree and for each split. Leaves with the same majority class were recursively merged with an m-value of two. The kNN approach used 10 nearest neighbors. The NN approach included a single hidden layer with 10 nodes, a regularization factor of one and 1,000 maximum iterations. The SVM approach used a radial basis function as a kernel. The accuracy of each classifier was returned. All machine learning analyses were implemented using the open-access Orange data mining software package that includes a variety of tools for manipulating data, performing machine learning analyses, and evaluating results [Demšar et al., 2013].

## Results

The biology-based framework and example liability distributions for Models 1–8 are shown as screenshots from the HIBACHI software in Figures4. Also shown next to each biological models is a screenshot from the MDR software summarizing the distribution of cases (dark bars) and controls (light bars) for each multilocus genotype. Genotype combination cells are dark-shaded for high-risk and light-shaded for low risk. Note the additive pattern of high-risk genotypes for Models 1–3. Also shown below each MDR figure is a screenshot from the ViSEN software showing the genetic variant interaction network for the two transcription factors (TF1, TF2) and the enhancer. The size of the circles representing each variant is proportional to the degree of main effect. Synergistic interactions are indicated by a line connecting two variants or a triangle connecting three variants with the thickness of each proportional to the degree of epistasis after removing lower order effects. Note the difference in distribution of main effects versus interaction effects in Models 1–3 and in Models 4–8.

**Figure 2:**
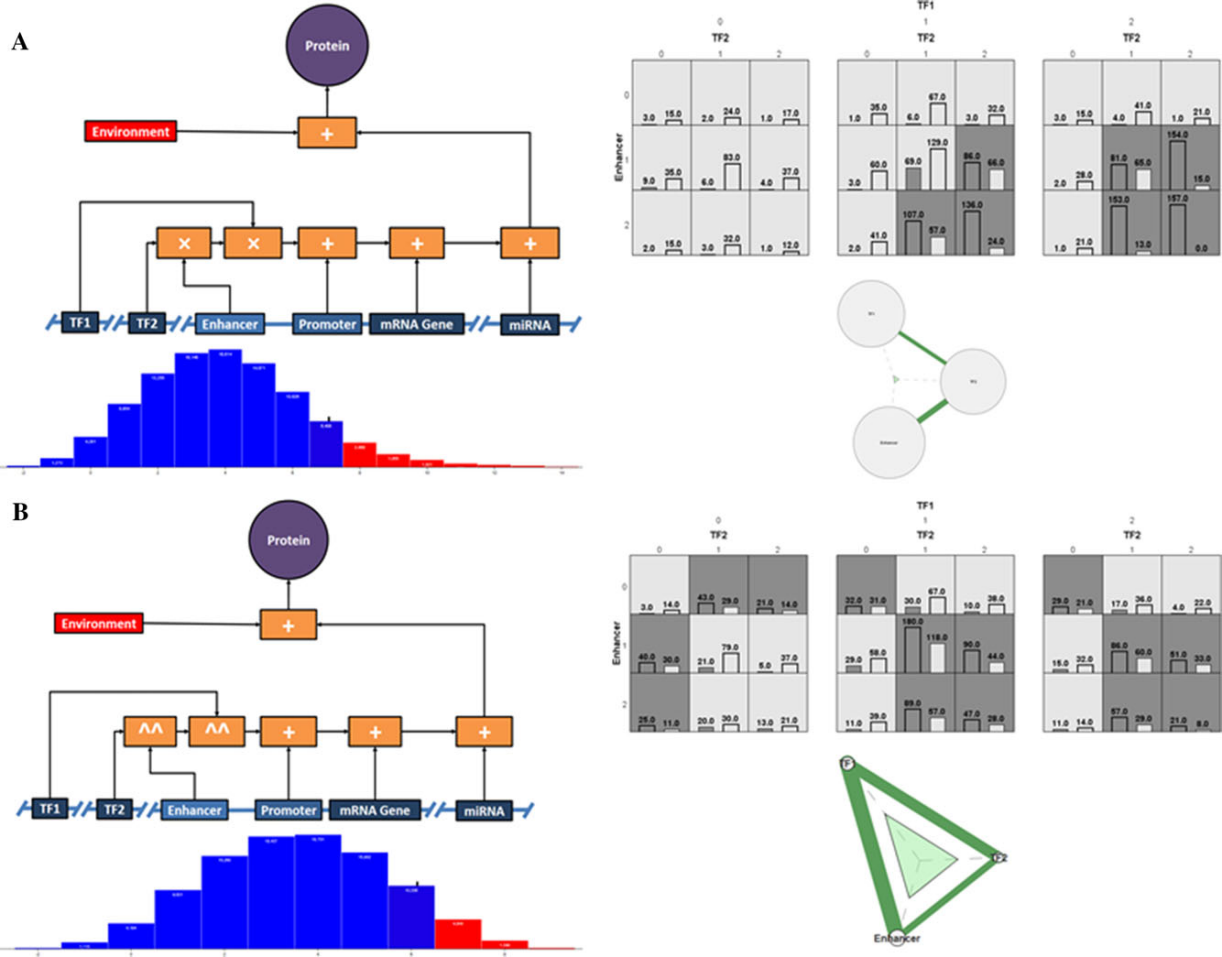
The left panels show screenshots of the biological and mathematical framework as well as the liability distribution for Models 3 (A) and 4 (B). The black notches on the right side of the liability distributions indicate the threshold for disease. To the right of each HIBACHI model is the MDR model showing the distribution of cases (dark bars) and controls (light bars) for each genotype combination. Dark-shaded genotype combinations indicate high-risk of disease. Shown below each MDR model is the ViSEN network with main effects (circles), pairwise interactions (lines), and three-way interactions (triangles) highlighted in proportion to their effect size.

**Figure 3:**
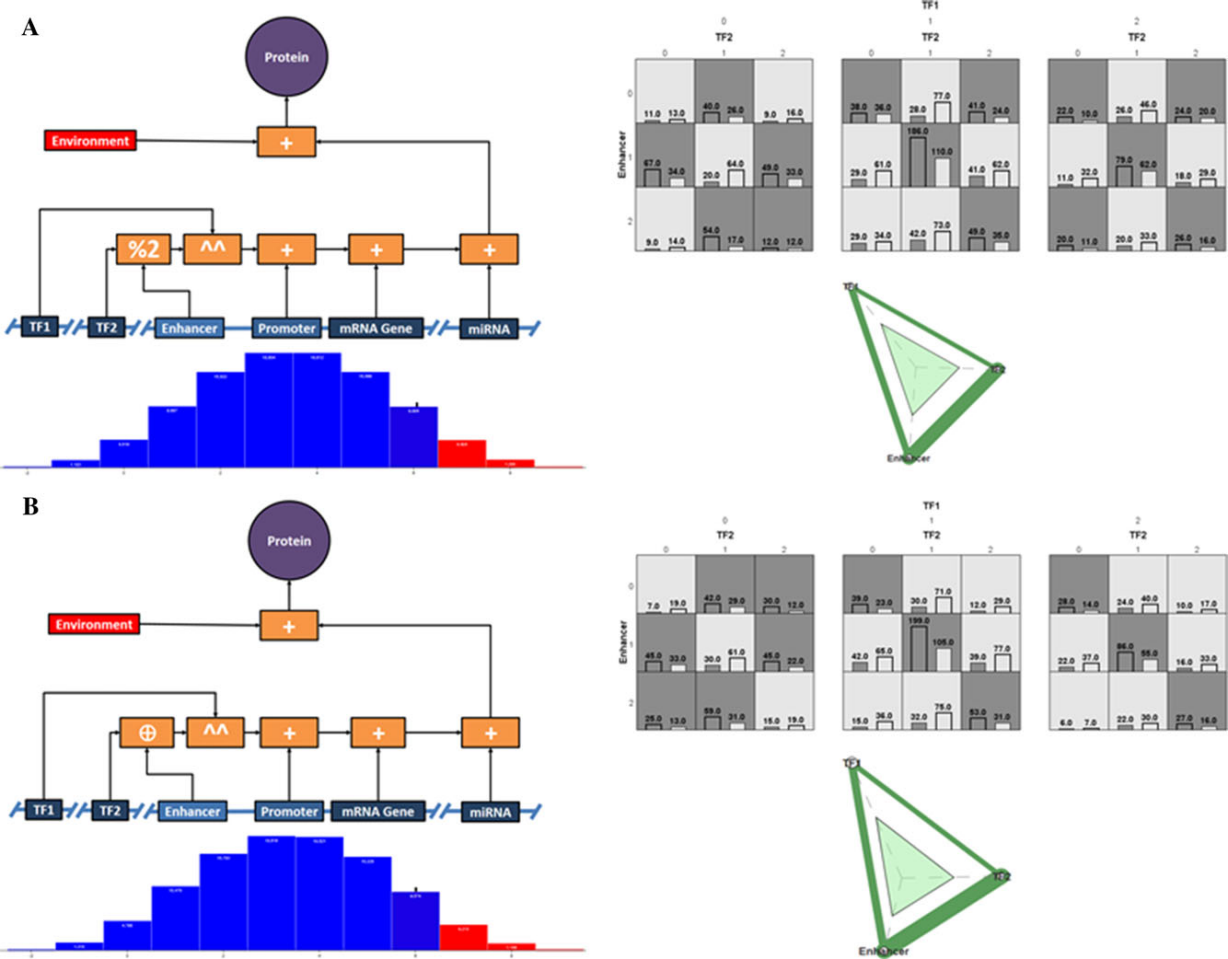
The left panels show screenshots of the biological and mathematical framework as well as the liability distribution for Models 5 (A) and 6 (B). The black notches on the right side of the liability distributions indicate the threshold for disease. To the right of each HIBACHI model is the MDR model showing the distribution of cases (dark bars) and controls (light bars) for each genotype combination. Dark-shaded genotype combinations indicate high-risk of disease. Shown below each MDR model is the ViSEN network with main effects (circles), pairwise interactions (lines), and three-way interactions (triangles) highlighted in proportion to their effect size.

**Figure 4:**
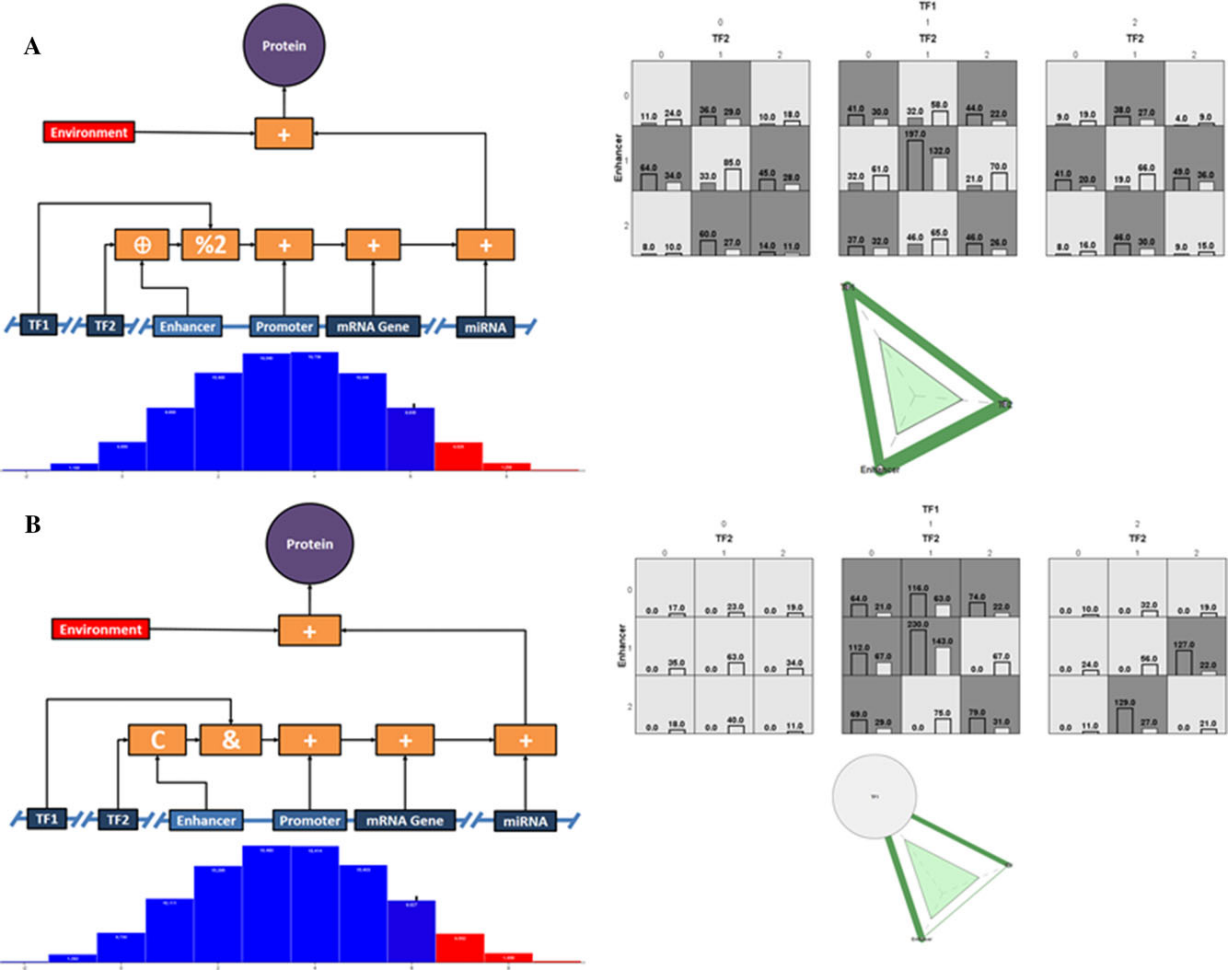
The left panels show screenshots of the biological and mathematical framework as well as the liability distribution for Models 7 (A) and 8 (B). The black notches on the right side of the liability distributions indicate the threshold for disease. To the right of each HIBACHI model is the MDR model showing the distribution of cases (dark bars) and controls (light bars) for each genotype combination. Dark-shaded genotype combinations indicate high-risk of disease. Shown below each MDR model is the ViSEN network with main effects (circles), pairwise interactions (lines), and three-way interactions (triangles) highlighted in proportion to their effect size.

Table1 presents the details of the eight models, the mathematical functions used for the three genetic variants, the information gain for each genetic variant alone (i.e. main effects), the information gain for the pure three-way interaction, the accuracy of the MDR classifier, the *P*-value of the MDR model from a standard permutation test, and the MDR *P*-value derived from a permutation test that explicitly tests for interaction holding all main effects constant. All eight models have MDR accuracies significantly above 0.5 ranging from 0.621 (moderate effect size) to 0.822 (large effect size). The models differ greatly with respect to their main effects and three-way interaction effects. As expected the Models 1–3 with only addition and/or multiplication functions score high on the information gain for main effects but very low for three-way interactions. The significant or near-significant *P*-values for the explicit permutation test are primarily due to some small to moderate two-way interactions (data not shown). Model 4–7 on the other hand have very low main effects and moderate three-way interactions with highly significant explicit tests of interaction. Model 8 was a bit different in that it contained a moderate main effect for one genetic variant in addition to a stronger three-way interaction. These eight models highlight the ability of HIBACHI to simulate a variety of different types of genetic effects including high-order epistatic interactions.

**Table 1:** Performance measures shown include information gain (IG) and classification accuracy. The first *P*-value shown is derived from a standard permutation test although the second comes from the explicit test of epistasis

Model	Functions	IG (TF1)	IG (TF2)	IG (Enhancer)	IG (TF1, TF2, Enhancer)	Accuracy	*P*-value	*P*-value
1	ADD, ADD	0.079	0.067	0.081	0.002	0.732	<0.001	0.06
2	ADD, MULT	0.303	0.103	0.102	0	0.822	<0.001	0.043
3	MULT, MULT	0.137	0.134	0.148	0.002	0.817	<0.001	<0.001
4	XOR, XOR	0.006	0.003	0.008	0.033	0.644	<0.001	<0.001
5	MOD2, XOR	0.002	0	0.001	0.046	0.621	<0.001	<0.001
6	BITX, XOR	0.003	0.001	0.001	0.04	0.644	<0.001	<0.001
7	BITX, MOD2	0	0	0.002	0.058	0.636	<0.001	<0.001
8	CHS, BITA	0.146	0.002	0.001	0.16	0.7875	<0.001	<0.001

**Figure 5:**
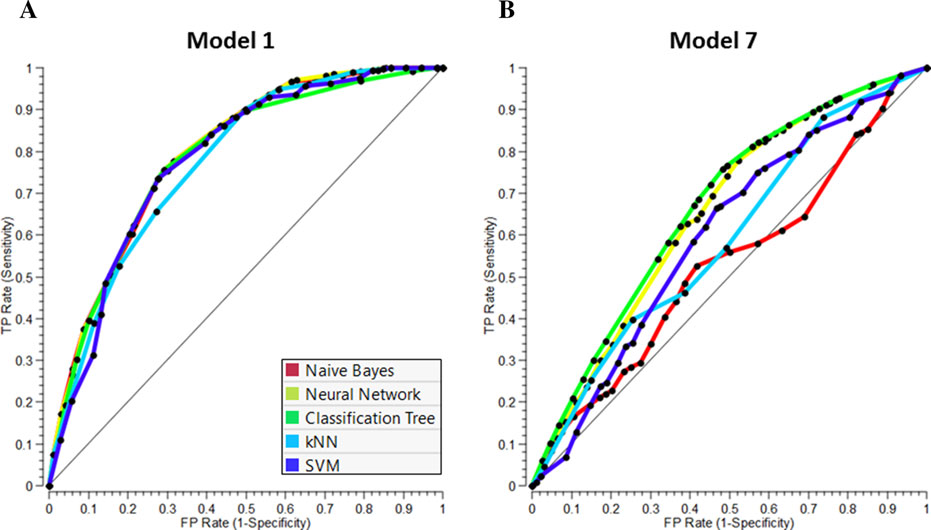
Receiver operating characteristic (ROC) curves for each machine learning method applied to example data sets from Models 1 (A) and 7 (B). Note the performance diversity of the different methods for Model 7 where the genetic effects are mostly due to three-way interactions.

Table2 presents the results of the machine learning algorithm comparison on example data from Models 1–8. The numbers shown are classification accuracies. Note that most of the methods were in close agreement for Models 1–3 but diverged across data from Models 4–8. This was particularly true for Models 4–7 that had virtually no main effects and complex patterns of three-way interactions. The NB approach performed poorly on these models given it is not designed to detect complex interactions. The diversity of results across the different models suggests that HIBACHI produces complex interactions that will be useful for comparing different methods for detecting complex genetic patterns. Figure5 shows example receiver operating characteristic (ROC) curves summarizing the balance between specificity (x-axis) and sensitivity (y-axis) for each machine learning method on an example data set from Model 1 and Model 7. Note that for a simple additive model each machine learning method has a similar area under the curve (AUC) of approximately 0.79. The model that includes a three-way epistatic interaction as the primary genetic effect yields a diversity of machine learning performance results with AUCs ranging from a low of 0.53 for the naïve Bayes method and a high of 0.67 for the classification tree. Again, this demonstrates the value of these models and the data simulated from them for comparing the performance of different machine learning methods on detecting different kinds of genetic effects.

**Table 2:** Machine learning method include classification trees (CT), k-nearest neibors (kNN), naïve Bayes (NB), neural networks (NN), and support vector machines (SVM). Numbers shown are classification accuracies

		Classification accuracy				
Model	Functions	CT	kNN	NB	NN	SVM
1	ADD, ADD	0.732	0.66	0.73	0.732	0.73
2	ADD, MULT	0.842	0.845	0.842	0.842	0.845
3	MULT, MULT	0.835	0.838	0.835	0.835	0.835
4	XOR, XOR	0.638	0.572	0.618	0.64	0.63
5	MOD2, XOR	0.625	0.575	0.488	0.618	0.59
6	BITX, XOR	0.625	0.59	0.555	0.625	0.568
7	BITX, MOD2	0.635	0.548	0.508	0.585	0.558
8	CHS, BITA	0.788	0.76	0.658	0.788	0.778

## Discussion

We have presented a prototype of a new biology-based method and software for simulating genotype–phenotype relationships in population-based data. This approach is different from other simulation methods in that it uses a hierarchical mathematical framework for mapping genotypic values from multiple loci to phenotypic values using gene structure and function as a scaffold. This will facilitate that simulation of genetic data that is more closely aligned with our understanding of how genetic variation impacts phenotypic variation through a hierarchy of biochemical mechanisms such as transcription and translation. This is important as it is becoming clear that the detection and interpretation of genetic associations in the context of genome-wide association studies (GWAS) and whole-genome sequencing (WGS) will depend on our understanding of genome biology including, for example, regulatory regions [Cowper-Sal lari et al., 2011; Karczewski et al., 2013]. We demonstrated this new method by generating several models with mostly independent main effects and several models with mostly three-way epistatic interactions. We characterized data simulated from each of these models with a variety of methods including MDR and several machine learning methods. The result suggests that this approach is capable of generating complex genotype–phenotype patterns that will be useful for the development and testing of new genetic analysis algorithms.

The approach presented here is a prototype that needs much more development. There are several important ways in which we envision modifying and extending the method and the software. First, we limited the initial simulation to six genetic variants from a single gene and some of its regulatory factors. This could be expanded significantly to include additional regulatory components and many more genetic variants within and across all the elements. For example, the mRNA gene could be broken down into introns and exons with multiple coding variants and noncoding variants that control mRNA splicing. The regulatory region could be expanded to include multiple variants in the promoter and enhancer with many transcription factor binding sites. Epigenetic variation could also be introduced. Enhancing the simulation is this way will expand the realism and complexity of a single locus. We also envision the protein product connecting with other protein products at a higher level through additional mathematical functions. The ability to include protein–protein interactions will allow for epistasis across protein-coding genes that extends beyond the regulatory networks described above. There is no reason why the simulation couldn't be expanded conceptually to cell–cell interactions and even tissue–tissue interactions that would introduce a systems-level component. Environmental and noise components would need to be incorporated into all levels of the hierarchy. These additional layers of complexity are needed to more closely mimic how real biological systems work.

An important limitation of the current study is that we did not consider effect size for each genetic variant. Our models included moderate to large genetic effects that are not realistic for common diseases. This can be addressed directly by changing the encoding on the genotypes. For example, we used 0, 1, and 2 to encode the different genotypes. This produces genotypes that contribute zero, one or two units of risk to the liability distribution depending of course on the mathematical function. These numbers could be divided by 10, for example, to reduce the risk contribution relative to the other genotypes. This would naturally reduce the effect size. Alternatively, we could introduce coefficients at the mathematical function layer. There is no reason why the result of any mathematical function could not be combined with a constant using a second function thus modifying its contribution to the liability. Either one or both of these mechanisms would allow the user to alter effect size. The challenge of course is that it might not be apparent how the effect size will change given the hierarchical complexity of the rest of the model. This could be greatly facilitated by perhaps specifying relative effect sizes for each genetic variant that could be used as a fitness objective in a heuristic search of the model space. We address new directions for the heuristic search next.

As the model complexity grows the heuristic search component will become more and more important as it becomes infeasible to develop models by hand due to the extreme number of parameters. We introduced here a simple random search and heuristic genetic algorithm that conducts a parallel stochastic search using the principles of natural selection. Other search algorithms such as simulated annealing will also be included in future versions thus allowing users to choose the search algorithm they think will be most effective. An element we think will be particularly effective for many different stochastic search algorithms is Pareto optimization [Coello et al., 2007; Horn et al., 1994]. This is a type of multiobjective optimization that allows the quality of models generated in a stochastic search to be evaluated by multiple different criteria that each are important to the user. Here, models are placed in *n*-dimensional space according to their values for *n* different criteria. The set of models for which there are no better according to all the criteria are selected. These models are referred to as nondominated and are said to exist on the Pareto front. The goal is to generate variability in the models through a method such as a genetic algorithm to move the Pareto front forward. This approach is advantageous because a model can be good for one criteria but perhaps not for others and still be retained. This allows the stochastic search algorithm to explore different criteria without penalty thus preserving the diversity of a search that might otherwise stall if only one criterion is used. This approach has worked well in the context of evolutionary search algorithms [Moore et al., 2013; Smits and Kotanchek, 2005]. Model criteria for HIBACHI could include, for example, the degree of interaction, effect sizes determined by coefficients, diversity of functions, normality or variance of the liability distribution, etc.

Finally, it will be important to develop an open-source and user-friendly software package that allows users to manually explore different models through a simple point and click interface such as that provided in Orange [Demšar et al., 2013] and through sophisticated stochastic search algorithms. The visual component will be important given this simulation approach is based on the hierarchical complexity of biological systems. We do not intend HIBACHI to be a comprehensive simulation package that produces patterns of genetic variations through methods such as forward-time simulation. Rather, we anticipate users will generate realistic genetic variation data with other existing packages and then will load selected variants into HIBACHI for the simulation of genotype–phenotype relationships. It is our working hypothesis that this biology-driven simulation approach and software will play an important role in the evaluation of new algorithms that embrace, rather than ignore, the complexity of genetic architecture and that make use of biology-driven data analysis methods such as gene-set enrichment approaches. It is time to move beyond simple statistical models for the both the simulation and the subsequent analysis of genetic data.

## References

[b1] Buchler NE, Gerland U, Hwa T (2003). On schemes of combinatorial transcription logic. Proc Natl Acad Sci USA.

[b2] Coello CAC, Lamont GB, Veldhuisen DAV (2007). Evolutionary Algorithms for Solving Multi-Objective Problems.

[b3] Collins RL, Hu T, Wejse C, Sirugo G, Williams SM, Moore JH (2013). Multifactor dimensionality reduction reveals a three-locus epistatic interaction associated with susceptibility to pulmonary tuberculosis. BioData Min.

[b4] Cordell HJ (2009). Detecting gene-gene interactions that underlie human diseases. Nat Rev Genet.

[b5] Cowper-Sal lari R, Cole MD, Karagas MR, Lupien M, Moore JH (2011). Layers of epistasis: genome-wide regulatory networks and network approaches to genome-wide association studies. Wiley Interdiscipl Rev Syst Biol Med.

[b6] Demšar J, Curk T, Erjavec A, Gorup Č, Hočevar T, Milutinovič M, Zupan B (2013). Orange: data mining toolbox in python. J Mach Learn Res.

[b7] Dudek SM, Motsinger AA, Velez DR, Williams SM, Ritchie MD (2006). Data simulation software for whole-genome association and other studies in human genetics. Pac Symp Biocomput.

[b8] Edwards TL, Bush WS, Turner SD, Dudek SM, Torstenson ES, Schmidt M, Ritchie MD (2008). Generating linkage disequilibrium patterns in data simulations using genomeSIMLA. Proceedings of the 6th European conference on Evolutionary computation, machine learning and data mining in bioinformatics.

[b9] Bernstein BE, Birney E, Dunham I, Green ED, Gunter C, Snyder M, ENCODE Project Consortium (2012). An integrated encyclopedia of DNA elements in the human genome. Nature.

[b10] Gerstein MB, Kundaje A, Hariharan M, Landt SG, Yan K-K, Cheng C, Snyder M (2012). Architecture of the human regulatory network derived from ENCODE data. Nature.

[b11] Greene CS, Himmelstein DS, Nelson HH, Kelsey KT, Williams SM, Andrew AS, Moore JH (2010). Enabling personal genomics with an explicit test of epistasis. Pac Symp Biocomput.

[b12] Hahn LW, Ritchie MD, Moore JH (2003). Multifactor dimensionality reduction software for detecting gene-gene and gene-environment interactions. Bioinformatics (Oxford, England).

[b13] Hernandez RD (2008). A flexible forward simulator for populations subject to selection and demography. Bioinformatics (Oxford, England).

[b14] Horn J, Nafpliotis N, Goldberg DE (1994). A niched Pareto genetic algorithm for multiobjective optimization.

[b15] Hu T, Andrew AS, Karagas MR, Moore JH (2013). Statistical epistasis networks reduce the computational complexity of searching three-locus genetic models. Pac Symp Biocomput.

[b16] Hu T, Chen Y, Kiralis JW, Collins RL, Wejse C, Sirugo G, Williams SM, Moore JH (2013). An information-gain approach to detecting three-way epistatic interactions in genetic association studies. J Am Med Inform Assoc.

[b17] Hu T, Chen Y, Kiralis JW, Moore JH (2013a). ViSEN: methodology and software for visualization of statistical epistasis networks. Genet Epidemiol.

[b18] Hu T, Chen Y, Kiralis JW, Moore JH (2013b). ViSEN: methodology and software for visualization of statistical epistasis networks. Genet Epidemiol.

[b19] Hu T, Sinnott-Armstrong NA, Kiralis JW, Andrew AS, Karagas MR, Moore JH (2011). Characterizing genetic interactions in human disease association studies using statistical epistasis networks. BMC Bioinformatics.

[b20] Karczewski KJ, Dudley JT, Kukurba KR, Chen R, Butte AJ, Montgomery SB, Snyder M (2013). Systematic functional regulatory assessment of disease-associated variants. Proc Nat Acad Sci USA.

[b21] Moore JH (2010). Detecting, characterizing, and interpreting nonlinear gene-gene interactions using multifactor dimensionality reduction. Adv Genet.

[b22] Moore JH, Asselbergs FW, Williams SM (2010). Bioinformatics challenges for genome-wide association studies. Bioinformatics (Oxford, England).

[b23] Moore JH, Boczko EM, Summar ML (2005). Connecting the dots between genes, biochemistry, and disease susceptibility: systems biology modeling in human genetics. Mol Genet Metab.

[b24] Moore JH, Gilbert JC, Tsai C-T, Chiang F-T, Holden T, Barney N, White BC (2006). A flexible computational framework for detecting, characterizing, and interpreting statistical patterns of epistasis in genetic studies of human disease susceptibility. J Theor Biol.

[b25] Moore JH, Hahn LW, Ritchie MD, Thornton TA, White BC (2004). Routine discovery of complex genetic models using genetic algorithms. Appl Soft Comput.

[b26] Moore JH, Hill DP, Sulovari A, Kidd LC, Riolo R, Vladislavleva E, Ritchie MD, Moore JH (2013). Genetic analysis of prostate cancer using computational evolution, pareto-optimization and post-processing. Genetic Programming Theory and Practice X.

[b27] Peng B, Amos CI (2008). Forward-time simulations of non-random mating populations using simuPOP. Bioinformatics (Oxford, England).

[b28] Peng B, Kimmel M (2005). simuPOP: a forward-time population genetics simulation environment. Bioinformatics (Oxford, England).

[b29] Purcell S, Neale B, Todd-Brown K, Thomas L, Ferreira MAR, Bender D, Sham PC (2007). PLINK: a tool set for whole-genome association and population-based linkage analyses. Am J Human Genet.

[b30] Ritchie MD, Bush WS (2010). Genome simulation approaches for synthesizing in silico datasets for human genomics. Adv Genet.

[b31] Ritchie MD, Hahn LW, Roodi N, Bailey LR, Dupont WD, Parl FF, Moore JH (2001). Multifactor-dimensionality reduction reveals high-order interactions among estrogen-metabolism genes in sporadic breast cancer. Am J Hum Genet.

[b32] Smits GF, Kotanchek M, O'Reilly U-M, Yu T, Riolo R, Worzel B (2005). Pareto-front exploitation in symbolic regression. Genetic Programming Theory and Practice II.

[b33] Tagkopoulos I, Liu Y-C, Tavazoie S (2008). Predictive behavior within microbial genetic networks. Science (New York, N.Y.).

[b34] Urbanowicz RJ, Kiralis J, Sinnott-Armstrong NA, Heberling T, Fisher JM, Moore JH (2012). GAMETES: a fast, direct algorithm for generating pure, strict, epistatic models with random architectures. BioData Min.

[b35] Walters RK, Laurin C, Lubke GH (2014). EpiPen: an R package to investigate two-locus epistatic models. Twin Res Hum Genet.

[b36] White MJ, Tacconelli A, Chen JS, Wejse C, Hill PC, Gomes VF, Sirugo G (2014). Epiregulin (EREG) and human V-ATPase (TCIRG1): genetic variation, ethnicity and pulmonary tuberculosis susceptibility in Guinea-Bissau and The Gambia. Genes Immun.

